# Novel Facet of an Old Dietary Molecule? Direct Influence of Caffeine on Glucose and Biogenic Amine Handling by Human Adipocytes

**DOI:** 10.3390/molecules26133831

**Published:** 2021-06-23

**Authors:** Wiem Haj Ahmed, Nathalie Boulet, Anaïs Briot, Barry J. Ryan, Gemma K. Kinsella, Jeffrey O’Sullivan, Francisco Les, Josep Mercader-Barceló, Gary T. M. Henehan, Christian Carpéné

**Affiliations:** 1 Institut des Maladies Métaboliques et Cardiovasculaires (I2MC), Institut National de la Santé et de la Recherche Médicale (INSERM U1048), I2MC, CEDEX 4, 31432 Toulouse, France; hajahmedwiem608@gmail.com (W.H.A.); nathalie.boulet@inserm.fr (N.B.); anais.briot@inserm.fr (A.B.); 2 Université Paul Sabatier, I2MC-UPS, CHU Rangueil, CEDEX 4, 31432 Toulouse, France; 3 School Food Science & Environmental Health, Technological University of Dublin, DOIWD85, D07 ADY7 Dublin, Ireland; barry.ryan@TUDublin.ie (B.J.R.); Gemma.Kinsella@tudublin.ie (G.K.K.); gary.henehan@TUDublin.ie (G.T.M.H.); 4 School of Dental Science, Trinity College, D02 PN40 Dublin 2, Ireland; josulli@tcd.ie; 5 Department of Pharmacy, Faculty of Health Sciences, Universidad San Jorge, Villanueva de Gállego, 50830 Zaragoza, Spain; fles@usj.es; 6 Molecular Biology and One Health (MOLONE) Research Group, Department of Fundamental Biology and Health Sciences, MOLONE, University of the Balearic Islands, 07120 Palma, Spain; josep.mercader@uib.es; 7 Balearic Islands Health Research Institute (IdISBa), 07120 Palma, Spain

**Keywords:** caffeine, adipocyte, lipolysis, lipogenesis, amine oxidases, methylxanthines, glucose transport

## Abstract

Caffeine is a plant alkaloid present in food and beverages consumed worldwide. It has high lipid solubility with recognized actions in the central nervous system and in peripheral tissues, notably the adipose depots. However, the literature is scant regarding caffeine’s influence on adipocyte functions other than lipolysis, such as glucose incorporation into lipids (lipogenesis) and amine oxidation. The objective of this study was to explore the direct effects of caffeine and of isobutylmethylxanthine (IBMX) on these adipocyte functions. Glucose transport into fat cells freshly isolated from mice, rats, or humans was monitored by determining [^3^H]-2-deoxyglucose (2-DG) uptake, while the incorporation of radiolabeled glucose into cell lipids was used as an index of lipogenic activity. Oxidation of benzylamine by primary amine oxidase (PrAO) was inhibited by increasing doses of caffeine in human adipose tissue preparations with an inhibition constant (Ki) in the millimolar range. Caffeine inhibited basal and insulin-stimulated glucose transport as well as lipogenesis in rodent adipose cells. The antilipogenic action of caffeine was also observed in adipocytes from mice genetically invalidated for PrAO activity, indicating that PrAO activity was not required for lipogenesis inhibition. These caffeine inhibitory properties were extended to human adipocytes: relative to basal 2-DG uptake, set at 1.0 ± 0.2 for 6 individuals, 0.1 mM caffeine tended to reduce uptake to 0.83 ± 0.08. Insulin increased uptake by 3.86 ± 1.11 fold when tested alone at 100 nM, and by 3.21 ± 0.80 when combined with caffeine. Our results reinforce the recommendation of caffeine’s potential in the treatment or prevention of obesity complications.

## 1. Introduction

Caffeine (1,3,7-trimethylxanthine) is a plant alkaloid found in food and beverages consumed daily worldwide. The mean daily intake of caffeine, was estimated to be 4 mg/kg of body weight in adults [1], and was more recently reassessed to average 165 mg (850 µmoles) per individual in the U.S. population [2]. Nowadays, many consumers of coffee, chocolate, and caffeinated beverages or adulterated food supplements [3] exceed this intake, approaching the no observable adverse effect level (NOAEL) for cardiovascular effects, established at 260 mg caffeine [4]. Due to its high lipid solubility, ingested caffeine is found in many tissues, i.e., brain, lung, liver, kidney, and adipose tissues [5]. Alongside its psychostimulant, anorexic, and somnolytic central actions, caffeine directly affects peripheral tissues, among them, the adipose depots. This study aims at exploring putative novel properties of caffeine that involve the role it plays in fat accumulation and body weight regulation. Caffeine has been considered as an anti-obesity drug [6], and has been found to interact directly with adipocytes or cultured preadipose cells in in vitro experiments at micromolar to supramillimolar levels.

In fat cells, lipogenesis and lipolysis are exquisitely regulated mechanisms that drive triacylglycerol accumulation and breakdown, respectively. Adipocyte lipogenesis corresponds to the incorporation of carbohydrates into lipids, mainly as a result of glycerogenesis and de novo synthesis of free fatty acids (FFA) from glucose, followed by triacylglycerol accumulation in lipid droplets. In post-prandial conditions, it is accompanied by FFA re-esterification, leading to the storage of circulating non-esterified fatty acids, except when the energy demand of the organism is increased (e.g., physical activity or cold exposure). While lipogenesis is activated by insulin, lipolysis (which involves the release of glycerol and FFA into the blood) is inhibited by insulin and activated by various lipolytic factors, including catecholamines. Caffeine facilitates lipolysis, by favoring the release of catecholamines via a central effect, and by increasing the adipocyte lipolytic response to ß-adrenergic receptor-mediated activation. In the case of lipolytic activation, the blockade of cAMP phosphodiesterases (PDE) and of several adenosine receptor types is the currently accepted mechanism of action of caffeine and other methylxanthines [6,7]. Both inhibitions lead to increased cAMP levels in fat cells. The same mechanism applies to the influence of caffeine on body weight control and thermogenesis [6,8,9,10].

Caffeine has also been reported to inhibit glucose transport in fat cells, which is the first step of lipogenesis [11,12,13]. However, it has been reported that several agents that increase cAMP levels in adipocytes, such as β-adrenergic agonists, can also increase glucose metabolism [14,15,16]. Consequently, the mechanisms involved in the limitation of glucose utilization by caffeine are likely cAMP-independent but remain elusive. A direct blockade of insulin-sensitive glucose carriers has been proposed for caffeine, as for other phytochemicals such as forskolin [11]. Despite these possible mechanisms for methylxanthine action, a number of recent observations prompted us to examine other pathways potentially involved in the antilipogenic responses to caffeine.

Adipocytes highly express amine oxidases such as monoamine oxidases (MAO) and copper-containing amine oxidases that are sensitive to semicarbazide (SSAO) [17]. SSAO is a multifunctional enzyme, renamed primary amine oxidase (PrAO), since it oxidizes a range of biogenic or exogenous amines and it is inhibited by numerous molecules besides semicarbazide [18]. SSAO/PrAO is highly expressed at the surface of adipocytes, smooth muscle cells, and inflamed blood vessels. Due to its role in the extravasation of leucocytes at the site of inflammation, it is also known as vascular adhesion protein-1 (VAP-1) [19]. SSAO/PrAO/VAP-1 is encoded by the gene *AOC3* (for copper-containing amine oxidase 3) [20]. We have reported that several inhibitors of PrAO, such as phenelzine, inhibit lipogenesis in rodent adipocytes [21,22], and proposed them as potential anti-obesity agents [23]. In addition, caffeine has been observed to inhibit both MAO [24] and PrAO [25,26], and recently these observations were extended to the amine oxidases expressed in human adipose tissue [27]. It was therefore tempting to hypothesize that inhibition of PrAO could contribute to the antilipogenic effect of caffeine. This reasoning was supported by Papukashvili et al. who proposed that inhibition of SSAO/PrAO by caffeine might contribute to its anti-obesity effects [28,29].

In this study, before examining the direct effects of caffeine on glucose utilization by fat cells, we re-assessed the short-term effects of isobutylmethylxanthine (IBMX) on lipolysis, glucose transport, and lipogenesis, in freshly isolated adipocytes, through a comparative analysis of hitherto unpublished data we obtained in rats, mice, and humans. In fact, the pharmacological agent IBMX has been used for decades in cell culture protocols and is well-recognized for promoting adipocyte differentiation. IBMX is used worldwide at 0.1–1 mM in in vitro adipogenic media, although it is not naturally occurring and is not found in food. IBMX has been more thoroughly studied in animal adipose-derived cells than in native human adipocytes and since our aim was to examine in humans the potential PrAO-dependent actions of methylxanthines, we took advantage of the observations accumulated in rodents and human with this agent before exploring caffeine’s effects on glucose handling in both animal and human fat cells. Thereafter, we compared the inhibitory effect of caffeine on lipogenesis in wild type (WT) mice and in mice bearing a mutation on the catalytic site of PrAO, which was obtained by a transgenic approach previously used to study the anti-inflammatory properties of VAP-1 [30]. These mice, called AOC3KI (for AOC3 knock-in), express a form of the gene *Aoc3* encoding a SSAO/PrAO/VAP-1 protein devoid of amine oxidase activity [30,31,32].

## 2. Results

### 2.1. Influence of IBMX on Lipolysis, Glucose Uptake and Lipogenesis in Rat, Mouse, and Human Adipose Cells

Before studying the short-term effects of caffeine on glucose utilization by fat cells, we examined another methylxanthine widely used in biochemical and pharmacological studies of cultured or freshly isolated adipocytes: the isobutylmethylxanthine (IBMX).

**Figure 1:**
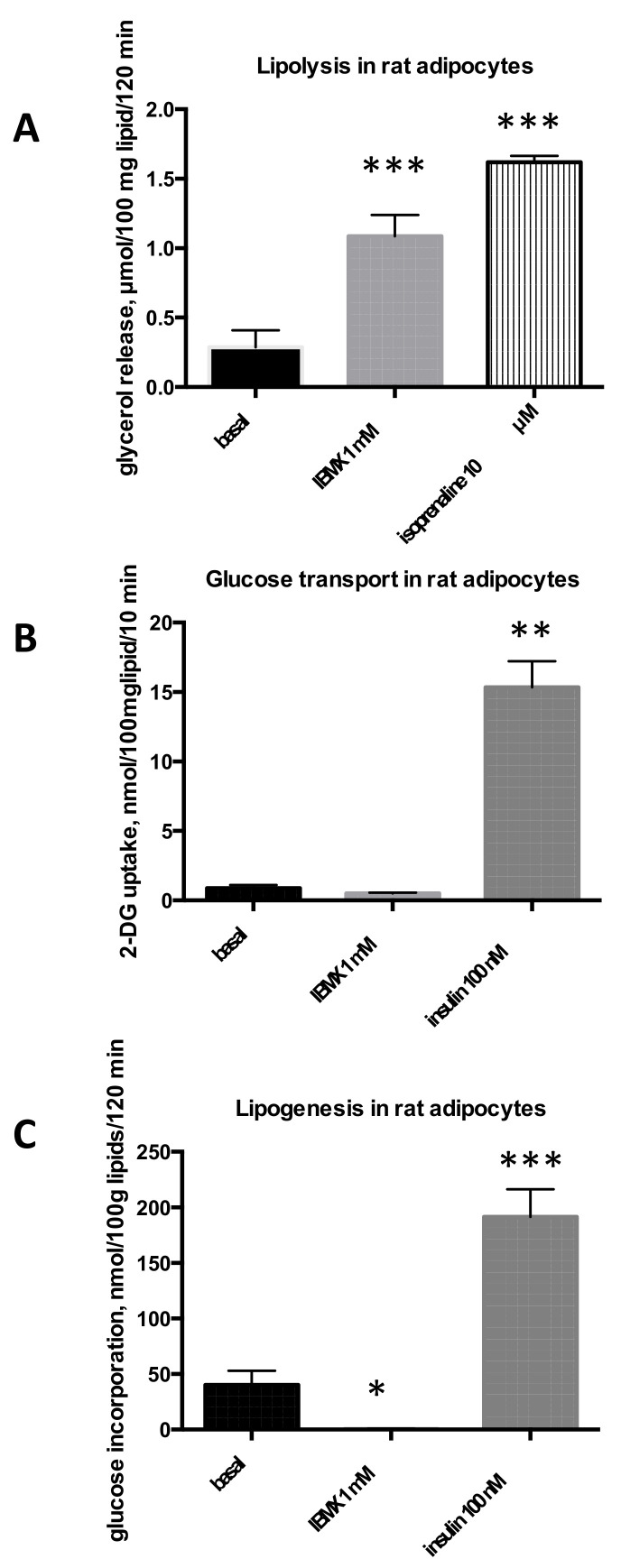
IBMX short-term effects on triacylglycerol breakdown and storage in rat adipocytes. Rat adipocytes were incubated without (basal, dark columns) or with 1 mM IBMX (grey columns) in the same conditions as the positive control (hatched or shaded columns), consisting of 10 µM isoprenaline for lipolysis (**A**), or 100 nM insulin for hexose uptake assay (**B**) and for determination of lipogenesis (**C**). The incubation period for IBMX or positive control with adipocytes was 120 min (**A,C**) or 45 + 10 min (**B**). Each column is mean ± SEM of 7 (lipolysis), 5 (glucose uptake), or 6 (lipogenesis) different determinations. Difference from respective basal at: * *p* < 0.05, ** *p* < 0.01, *** *p* < 0.001 by one-way ANOVA followed by Dunnett multiple comparisons.

As expected, IBMX activated lipolytic activity four-fold in rat adipocytes (Figure 1A). Further, 1 mM IBMX was almost as active as the β-adrenergic pan-agonist isoprenaline, which is the molecule of reference among lipolytic agents. When we examined the uptake of [^3^H]-2-deoxyglucose (2-DG) into rat adipocytes, insulin, the activator used as a reference, was clearly stimulatory. This was not the case for IBMX, which exhibited a tendency to inhibit basal uptake (Figure 1B). A similar pattern was observed when lipogenesis was measured: 100 nM insulin induced an almost six-fold increase in basal D-3-[^3^H]-glucose incorporation into intracellular lipids while 1 mM IBMX abolished lipogenic activity (Figure 1C).

**Figure 2:**
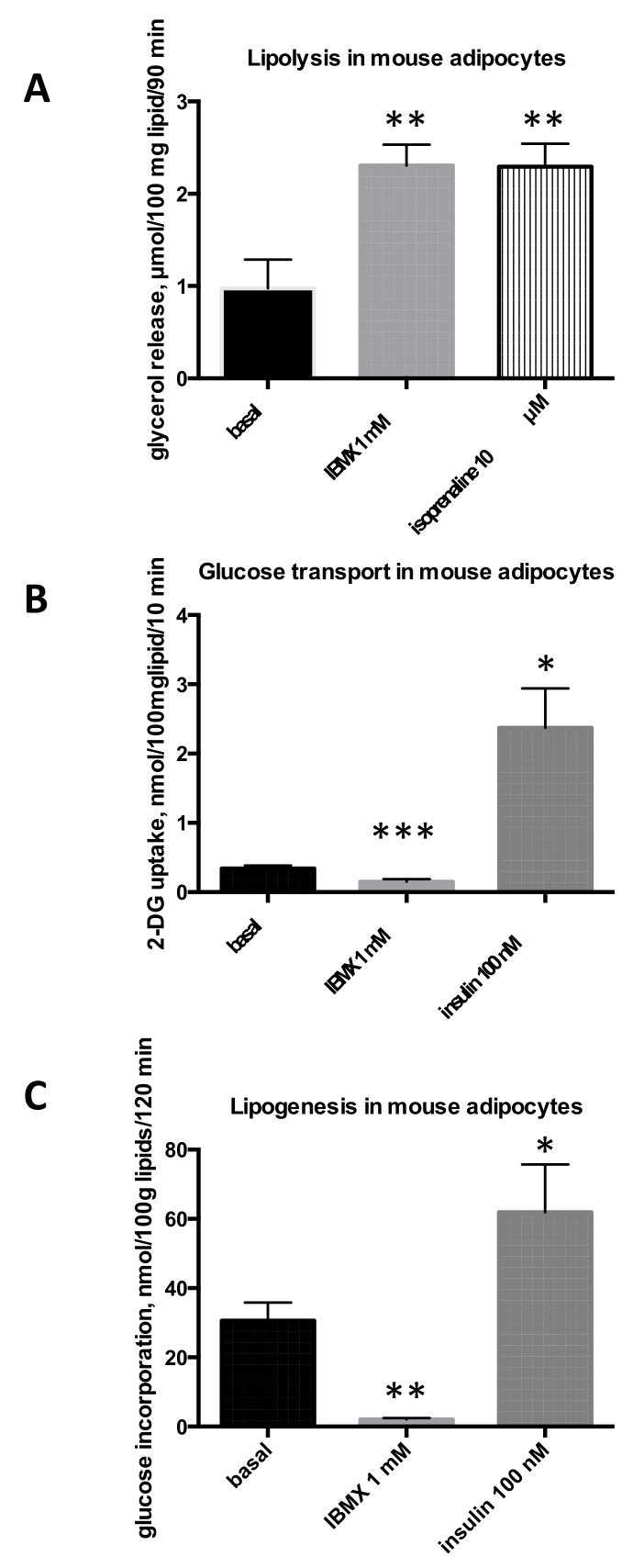
IBMX effects on triacylglycerol breakdown and storage in mouse adipocytes. Each column is the mean ± SEM from seven preparations for lipolysis (**A**), five preparations for hexose uptake (**B**), and eight adipocyte preparations for lipogenesis (**C**). Conditions were the same as in Figure 1, except that glycerol release was determined after 90 min incubation. Significantly different from each corresponding basal (black column) at: * *p* < 0.05, ** *p* < 0.01, *** *p* < 0.001.

In mouse adipocytes, similar responses were obtained: a lipolytic stimulation by both isoprenaline and IBMX (Figure 2A), while insulin and IBMX exerted opposite effects on glucose transport (Figure 2B) and incorporation into lipids (Figure 2C).

**Figure 3:**
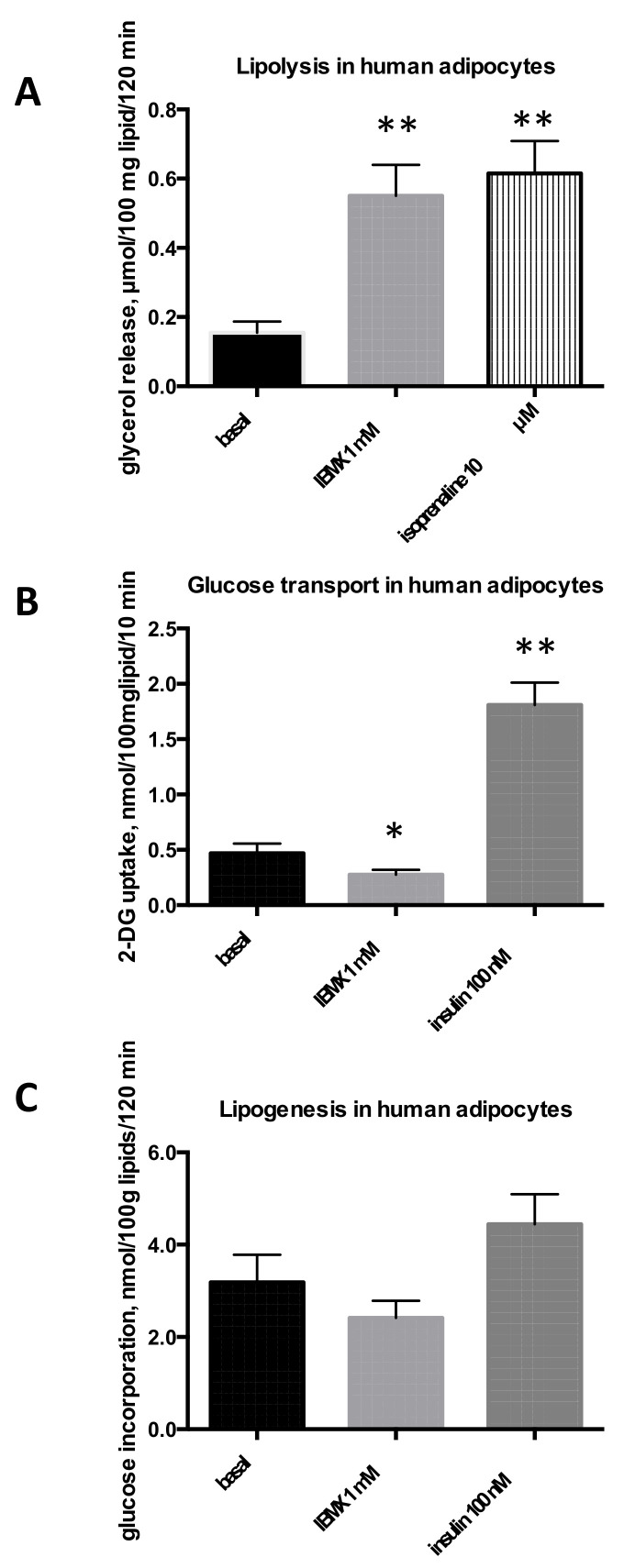
IBMX effects on triacylglycerol breakdown and storage in human adipocytes. Human adipocytes were isolated from subcutaneous adipose tissue obtained from a total of 18 adults undergoing abdominal dermolipectomy (mean age: 43; BMI = 26.0 ± 0.9) and tested for their lipolytic (**A**), glucose transport (**B**), or lipogenic (**C**) activities. As with rodent fat cells, basal levels were determined without any addition, while positive controls were: 10 µM isoprenaline for lipolysis or 100 nM bovine insulin for hexose uptake and lipogenesis. Each column is the mean ± SEM from six individuals. Significantly different from respective basal at: * *p* < 0.05, ** *p* < 0.01.

The same metabolic responses were explored in adipocytes freshly isolated from human subcutaneous adipose depots. As in rodents, IBMX stimulated lipolysis in human adipocytes. At 1 mM, it stimulated glycerol release to the same levels as 10 µM isoprenaline (Figure 3A). Additional observations indicated its activation of lipolysis was dose dependent, since at 10 µM and 100 µM, it induced 34 ± 8 and 72 ± 7% of the maximal response seen with isoprenaline (not shown). At 1 mM, IBMX reached 85 ± 7% of the isoprenaline effect (*n* = 6). IBMX also inhibited 2-DG uptake (Figure 3B). However, in these studies, human adipocytes were less responsive to insulin stimulation of hexose transport than rodent adipocytes; the respective stimulation magnitude being 25.5 ± 7.5, 6.9 ± 1.4, and 4.2 ± 0.6 fold increase over basal 2-DG uptake in rats, mice, and humans, respectively. This interspecies difference was even more evident for de novo lipogenesis, since insulin stimulated the basal incorporation of radiolabeled glucose into lipids by only 1.5-fold in human adipocytes (Figure 3C). Consequently, in our sample (*n* = 6 adults), no significant lipogenic effect could be detected for insulin, while for IBMX, there was only a tendency to inhibit basal lipogenesis.

Although some interspecific variation was observed, this comparative approach confirmed unequivocally that IBMX is a strong lipolytic agent, but also that IBMX directly impairs both hexose transport and glucose incorporation into adipocyte lipids. Only the latter inhibition could not be clearly evidenced in human adipocytes, which exhibit a very low spontaneous and stimulated lipogenic activity, at least when using glucose as substrate (compare Figure 1C, Figure 2C, and Figure 3C).

Taking advantage that rat adipocytes were the most responsive to insulin, we observed in additional experiments that IBMX not only lowered the baseline of glucose metabolism (see Figure 1B) since it rapidly and dramatically impaired the stimulation of glucose handling by insulin. When basal 2-DG uptake in rat adipocytes was arbitrarily set at 1.0, it was increased up to 25.5 ± 7.5 times in response to 100 nM insulin, while in the presence of insulin plus 1 mM IBMX, the increase was limited to only 4.3 ± 2.0 (*n* = 6, *p* < 0.003, not shown).

Consequently, and as generally the case for biological systems, it appears to be preferable to test the putative inhibitory effect of IBMX or any other agent on a pre-stimulated state than on a non-stimulated baseline. Therefore, it was of interest to explore whether this inhibitory property applied for another, much more nutritionally relevant methylxanthine, namely caffeine.

### 2.2. Effects of Caffeine on Glucose Uptake and Lipogenesis in Murine Adipose Cells

Considering that, as for IBMX, caffeine lipolytic effects are well documented in adipocytes [33,34,35] and since we recently reported that caffeine hampers the antilipolytic effect of insulin in mouse adipocytes [36], we focused our attention on how caffeine was able to impair insulin stimulation of glucose uptake and incorporation into lipids. In fact, caffeine clearly inhibited the insulin activation of 2-DG uptake in mouse adipocytes: when the response to 100 nM insulin, which resulted in a 10.3 ±1.4 fold increase over baseline, was set at 100%, only 55 ± 13% of this response was observed with 0.1 mM caffeine plus insulin (*n* = 4, *p* < 0.01, not shown).

**Figure 4:**
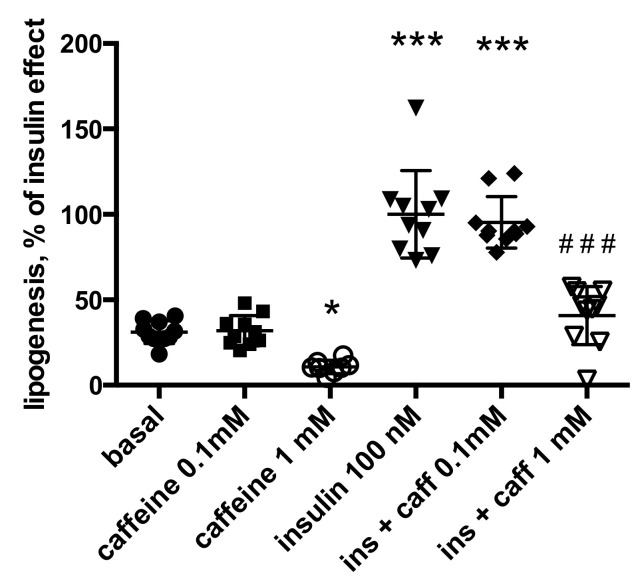
Caffeine inhibits basal and insulin-stimulated lipogenesis in mouse adipocytes. Data expressed as percent of maximal lipogenic response to insulin. The 100% indicates the mean glucose incorporation into lipids stimulated by insulin 100 nM found in ten different preparations of mouse adipocytes. Each condition was successfully tested in 10 adipocyte preparations, save for caffeine 1 mM alone (open circles), for which *n* = 8. Significance was determined compared to basal lipogenic activity (control, black circles) at: * *p* < 0.05, *** *p*< 0.001. Significant difference from insulin without caffeine (ins, inverted triangles) at: ^###^ *p* < 0.001).

Likewise, the lipogenic effect of insulin was inhibited by caffeine (Figure 4). In that case, 1 mM caffeine was required to totally abolish the response of mouse adipocytes to insulin, while at 0.1 mM, caffeine did not impair insulin-stimulated lipogenesis. The basal glucose incorporation into lipids was decreased by caffeine in a like manner (Figure 4). All of these rapid inhibitory effects by caffeine were essentially similar to the observations reported above for IBMX.

### 2.3. Is Caffeine Inhibiting Insulin Stimulation of Adipocyte Anabolism in Humans?

**Figure 5:**
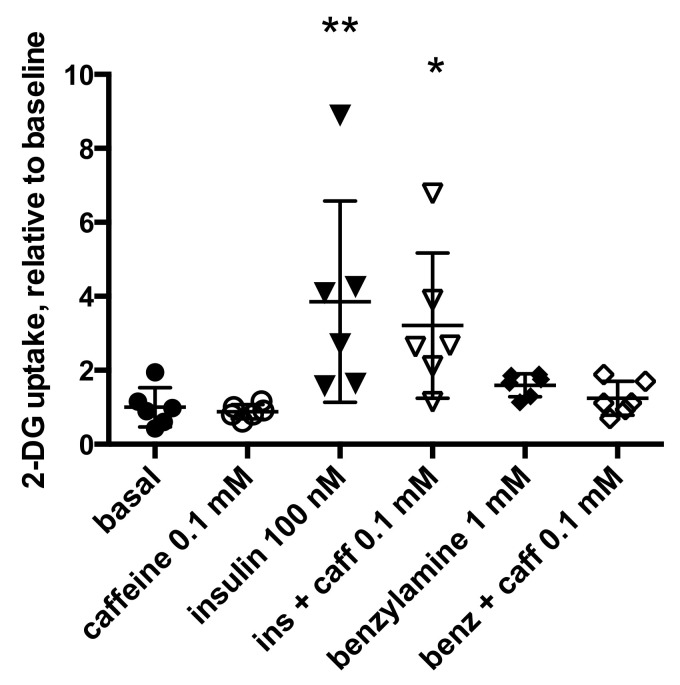
Influence of caffeine on [^3^H]-2-DG uptake by human adipocytes under basal conditions and when activated by insulin or benzylamine. Each agent was incubated 45 min with human adipocytes prior the determination of [^3^H]-2-DG uptake on a 10-min period. Each column is mean ± SEM of six individual preparations. Significant differences from basal 2-DG uptake (closed circles) at: * *p* < 0.05, ** *p* < 0.01. No significant effect of 0.1 mM caffeine (caff, open symbols) was found for each of the conditions tested: basal, insulin (ins), or benzylamine (benz).

Caffeine influence on hexose uptake was studied in human subcutaneous adipocytes under both basal and stimulated conditions. A tendency to reduce basal uptake was detected with 0.1 mM caffeine: relative to basal 2-DG transport, set at 1.00 ± 0.20 for six individuals, caffeine slightly reduced uptake to 0.83 ± 0.08. Insulin-stimulated transport gave 3.86 ± 1.11 times the baseline uptake using 100 nM insulin (*p* < 0.01), this value was reduced to 3.21 ± 0.80 (*p* < 0.05) when insulin was combined with caffeine (Figure 5).

**Figure 6:**
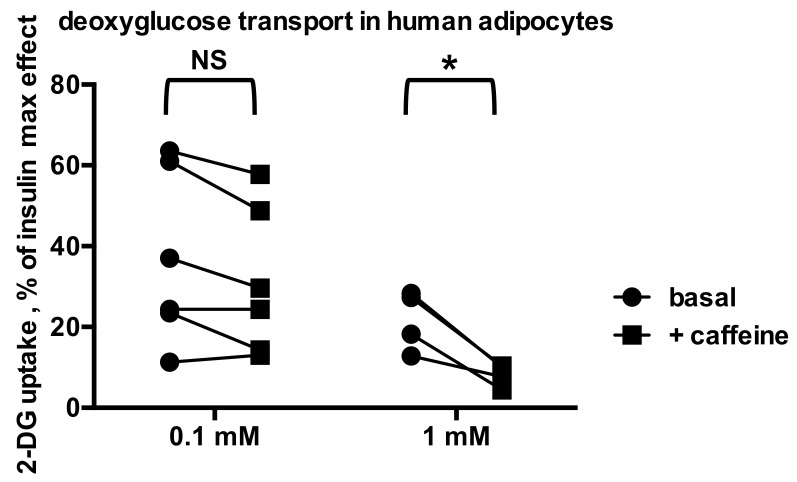
Influence of caffeine on basal hexose transport in human adipocytes. [^3^H]-2-DG uptake is expressed as percentage of maximal response to 100 nM insulin, set at 100% in each individual. Each couple of points corresponds to an adipocyte preparation from an adult individual, incubated without (basal, circles) or with the indicated dose of caffeine (squares). Difference from corresponding basal by paired *t* test. NS indicates differences that were not significant (data for six individuals) while * indicates significant inhibition (data for four individuals) at: *p* < 0.05.

Since millimolar doses of the PrAO substrate benzylamine have been reported to activate glucose transport in human adipocytes [37], we tested whether caffeine could impair this stimulation. In the six preparations studied, the effect of 1 mM benzylamine failed to double the baseline (1.60 ± 0.13 fold increase, NS) and no significant impairment was observed with 0.1 mM caffeine (Figure 5). We further observed in four other individuals that 1 mM caffeine lowered 2-DG uptake baseline in a manner that was significant after paired comparison, while 0.1 mM caffeine did not significantly alter basal hexose transport (Figure 6).

The planned experiments aiming to determine the influence of caffeine on insulin-induced lipogenesis in human adipocytes were discontinued since, as reported in the experiments shown in Figure 3 and those previously published [36], insulin did not significantly activate glucose incorporation into lipids in the four first cases studied (1.27 ± 0.18 increase over baseline, NS), preventing the detection of any possible inhibition. In addition, the amount of radioactivity incorporated in lipids was too limited for accurate data processing: averaging 897 disintegrations per min (dpm) for baseline and 1048 dpm for insulin, while background was at 134 dpm in these four individuals. It is worth mentioning here that, under similar conditions, basal [^3^H]-glucose incorporation into rat adipocyte lipids averages 4900 dpm and can be increased by between five to 25 times in response to insulin.

As indicated above, human fat cells appear therefore to be less metabolically active than the rodent ones. Fatty acid synthesis from glucose is not a major pathway for triacylglycerol accumulation in human adipose tissue, which instead uses re-esterification pathways and circulating lipids as a source for fat accumulation. As we could not use larger amounts of human biological resource or higher levels of radiolabeled precursors to solve these technical issues, we further explored whether caffeine was able to inhibit PrAO activity in human adipose tissue.

### 2.4. Inhibition by Caffeine of PrAO and MAO Activities in Human Adipose Tissue

**Figure 7:**
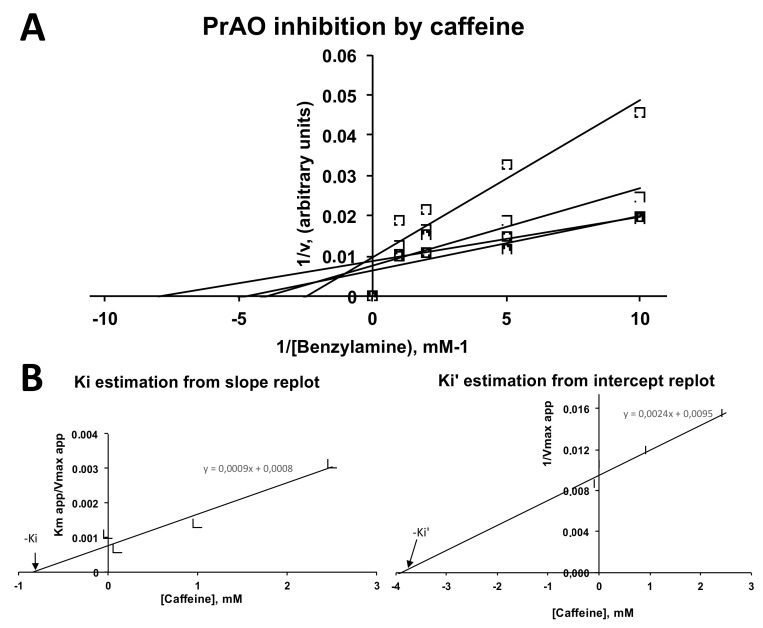
Caffeine inhibits PrAO in human adipose tissue. (**A**) Lineweaver–Burk plot of the inhibition of benzylamine oxidation by increasing doses of caffeine at 0.1, 1.0, and 2.5 mM. Each point is the mean of four to 10 determinations of hydrogen peroxide release over 30 min by homogenates of subcutaneous adipose tissue. (**B**) Replots from the slopes (left, Ki) or the intercepts (right, Ki’) of the double reciprocal plots.

Human adipose tissue readily oxidized tyramine and benzylamine as recently reported [27]. The inhibition of human MAO by caffeine, already described for recombinant enzymes by Petzer et al. [24], was confirmed in human adipose tissue preparations: Ki was 0.33 mM and Ki’ was 1.8 mM (mixed inhibition; data not shown). Benzylamine oxidation by human adipose tissue preparations is totally abolished by semicarbazide inhibition as already reported [37], therefore due solely to SSAO/PrAO, and was impaired in the presence of caffeine (Figure 7). This was in agreement with previous observations of caffeine inhibition of human PrAO [27]. In the concentration range 0.1–2.5 mM, caffeine inhibited human PrAO with a broadly mixed pattern of inhibition. Ki estimation for the inhibition by caffeine was 0.88 mM while Ki’ was 3.95 mM, in close similarity to the parameters previously found with bovine soluble plasma amine oxidase [26].

The potency of caffeine inhibiting benzylamine oxidation by human adipose tissue was in good agreement with its capacity to limit basal or insulin-stimulated glucose consumption by human adipocytes. Both inhibitory activities required millimolar doses, as found for other biological short-term or long-term responses to caffeine [34], including its lipolytic effect [35].

### 2.5. Exploring the Mechanisms Involved in Caffeine Inhibition of Lipogenesis

On the one hand, it has been recently proposed that the inhibition of PrAO/SSAO by caffeine may contribute to its weight gain attenuation alongside its inhibition of adipocyte cAMP PDE [28,29]. On the other hand, we have reported that several pharmacological PrAO blockers such as phenelzine inhibit adipocyte lipogenesis [21]. Thus, it was tempting to investigate whether PrAO inhibition was involved in the inhibitory effect of caffeine on insulin-stimulated glucose utilization.

We recently reported that AOC3KI mice, genetically invalidated for the *Aoc3* gene encoding for SSAO/PrAO/VAP-1, exhibit a moderate obesity, together with a total abolition of PrAO activity, notably in adipose tissues [32]. AOC3KI adipocytes express the PrAO protein but it is enzymatically inactive due to an engineered mutation in the active site. Investigating whether a disappearance of the antilipogenic action of caffeine occurred in the adipocytes of AOC3KI model appeared therefore highly appropriate.

**Figure 8:**
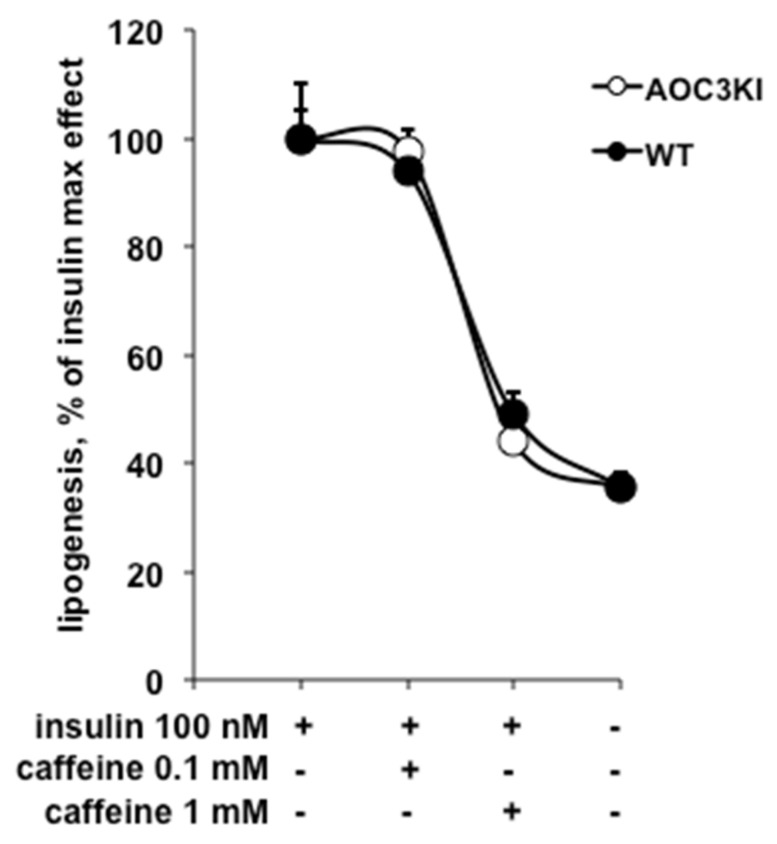
Caffeine impairs insulin-stimulated lipogenesis in mouse adipocytes irrespective of their SSAO/PrAO activity level. Insulin-stimulated lipogenesis was determined via the incorporation of [^3^H]-glucose into lipids of adipocytes freshly isolated from wild-type (WT, black circles) or SSAO/PrAO-invalidated mice (AOC3KI, open circles) measured after 120 min incubation with 100 nM insulin. Lipogenesis is expressed on the Y-axis as percentage of glucose incorporation into adipocyte lipids in response to 100 nM insulin. The X-axis displays the following conditions: insulin, insulin with caffeine at two concentrations, basal. Each point is the mean ± SEM of 8–14 mice. No significant effect of SSAO/PrAO invalidation was detected on the antilipogenic effect of caffeine, which, at 1 mM, was significant at *p* < 0.001 in both genotypes.

**Figure 9:**
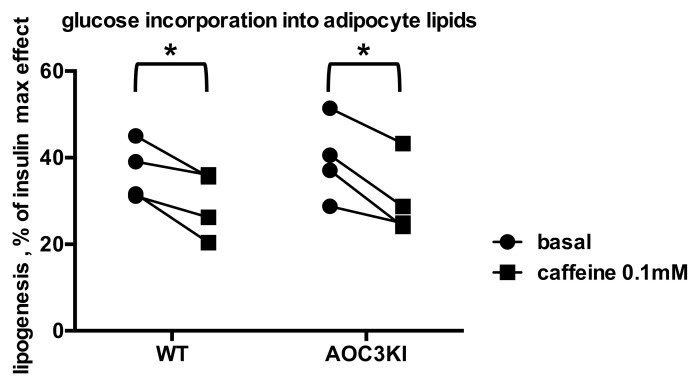
Caffeine inhibits basal lipogenesis in mouse adipocytes irrespective of their SSAO/PrAO activity level. Lipogenesis is expressed as percentage of maximal activity, with 100% corresponding to glucose incorporation into lipids in response to 100 nM insulin. Each couple of points corresponds to a preparation of adipocytes from wild type (WT) or transgenic (AOC3KI) mouse, incubated without (basal, circles) or with 0.1 mM caffeine (squares). Different from corresponding basal by paired *t* test at: * *p* < 0.05.

Surprisingly, there was no significant difference between AOC3KI and wild type (WT) mice regarding the antilipogenic effect of caffeine, which hampered the insulin stimulation, in a dose-dependent manner in both genotypes (Figure 8). This similarity was not an artificial consequence of putative changes in insulin responsiveness since basal lipogenesis was increased by 2.9 ± 0.2 times in WT and 2.7 ± 0.3 in AOC3KI mice (NS). In addition, we noticed that, even in the absence of insulin, the baseline levels of radiolabeled glucose incorporation into lipids were lowered by caffeine in the two genotypes. Paired comparison indicated that 1 mM caffeine lowered the lipogenic activity in each mouse adipocyte preparation studied, irrespective of its PrAO activity level (Figure 9). Similarly, the MAO inhibitor pargyline remained unable to substantially inhibit the insulin-stimulated lipogenesis in both genotypes since 88 ± 7% and 89 ± 2% of insulin effect resisted to the presence of 1 mM pargyline (data not shown). The only change noted between WT and AOC3KI mice was that the stimulation of glucose uptake and incorporation into lipids by the PrAO substrate benzylamine was reduced when PrAO was invalidated, as we recently documented in [32]. Taken together, the data obtained with the AOC3KI model indicated that the antilipogenic action of caffeine was independent of PrAO inhibition, at least in mice.

## 3. Discussion

In the present work, no effort was made to re-examine the lipolytic effect of caffeine in adipocytes since it is widely recognized that it is mediated by the stimulation of Gi-coupled purinergic receptors and PDE inhibition, both raising cAMP levels, and thus protein kinase A and hormone sensitive lipase [38]. With this in mind, we mention that the simple removal of adenosine from the incubation medium of rat adipocytes, obtained by adding adenosine deaminase at 0.4 IU/mL, produced the same lipolytic response as 1 mM IBMX (1.08 ± 0.15 vs. 1.09 ± 0.13 µmoles of glycerol released/100 mg cells/10 min, respectively, *n* = 6, NS). However, IBMX is a potent antilipogenic agent while adenosine deaminase is not. This prompted us to hypothesize that, aside from their lipolytic effect, methylxanthines influence glucose transport and incorporation into lipids (known as de novo lipogenesis) independently from their actions on cAMP levels, and differently from other lipolytic agents. Since we previously observed that the PrAO/MAO non selective inhibitor phenelzine is antilipogenic [21] and considering that methylxanthines inhibit both PrAO and MAO [18,25,26,27], our attention was directed to investigate whether PrAO inhibition was a mechanism necessary and sufficient for caffeine inhibition of glucose metabolism, as suggested in a recent review by Papukashvili et al. [28]. Three other findings supported this assumption: (1) PrAO substrates, such as benzylamine and methylamine stimulate glucose transport in human adipocytes [37,39]; (2) another phytochemical, resveratrol, is able to inhibit both MAO and lipogenic activities in mouse fat cells [40]; (3) as for caffeine, pharmacological inhibitors of SSAO have been proposed as potential anti-obesity agents [23]. Although the main objective of the present work was to demonstrate that PrAO inhibition participates in the antilipogenic action of caffeine, our findings indicated that PrAO inhibition by caffeine occurs in adipose cells but is apparently not essential for caffeine’s antilipogenic effects. Hence, the characteristics of these two in vitro effects and whether they are of nutritional or toxicological relevance is discussed below.

First of all, the observed inhibition of glucose metabolism and of amine oxidase activity reported here for caffeine could not be considered as artefactual. Since caffeine was readily soluble in water at 10 mM, the use of vehicles such as DMSO or ethanol to dissolve the substance was not necessary, as it was the case for other naturally occurring methylxanthines, for which vehicle effects needed corrections and were far from negligible [27].

Regarding the inhibition of amine oxidases, the inhibition constants (Ki) that measure the affinity of caffeine for its putative targets, were obtained in human adipose tissue homogenates, which means in the presence of the native form of the enzymes, surrounded by a myriad of intra- and extra-cellular components (such as lipids, metabolites, ions, modulators) that might potentially affect the access of substrates and inhibitors to the catalytic site or alter the enzymes in an allosteric manner. The use of purified enzyme preparations would have generated more accurate values of kinetic constants and better definition of the nature of inhibition by caffeine. This would have resulted in potentially lower Ki, but it must be taken into account that all these interfering components present in the crude preparations we studied are likely playing a role in the reality of the interactions of ingested caffeine with its multiple targets within the organism. Thus, based on these somewhat ‘’crude’’ Ki values for human MAO and PrAO inhibition, which are in the millimolar range, one can conclude that the inhibition of amine oxidases by caffeine occurs at larger concentrations than those required for other known targets. By comparison, the Ki for caffeine is around 40 µM for the antagonism at adenosine receptors and 480 µM for the PDE inhibition (reviewed in [41]). However, caffeine accumulates in adipose depots, as observed in rats [5]. Che et al. observed that, in rats receiving 50 mg caffeine /kg /day, i.p., the caffeine level was limited to 0.07 µg/mg of adipose tissue after 10 days, and increased to 0.22 µg/mg after 25 days of administration. More importantly, the caffeine inhibitory effect on PrAO activity was most effective in adipose tissue than in other organs. It was equivalent to a two-third inhibition compared to control, indicating that in vivo, the PrAO highly expressed in adipose tissue [32] is one of the main targets of caffeine [5]. Moreover, a partial, although incomplete, inhibition of PrAO by caffeine could be reinforced by other dietary phytochemicals or their metabolites that often accompany caffeine in beverages, and can be sufficient to modulate PrAO and its related biological roles. One must consider that pharmacological inhibitors of PrAO/VAP-1 are developed as anti-inflammatory agents [19,20] and that adipose tissue has been already implicated in the broad immunomodulatory actions of caffeine [42] since the methylxanthine inhibits the expression of the proinflammatory cytokine TNFα [43]. Whether a partial inhibition of PrAO/VAP-1 contributes to the anti-inflammatory actions of caffeine [8,42] or to substantially reduce the oxygen reactive species generated by oxidative deamination remains to be determined.

Regarding the inhibition of insulin-induced glucose utilization, our results indicate that it was obtained after relatively short-term exposure (from 45 to 120 min) and confirmed the long-term effects of caffeine and its metabolites on cultured preadipocytes [13] or mature rodent adipocytes [36]. An impairment of insulin responses was observed essentially at millimolar doses of caffeine and can be considered to occur at the frontier between pharmacology and toxicology. First, it should be noted that 2.5 mg caffeine/kg body weight remains an appropriate recommendation for daily ingestion in healthy individuals [44], which is roughly equivalent to 10 µmol/kg. Moreover, many pharmacokinetics studies performed with caffeine or caffeine-containing beverages have reported human plasma Cmax levels varying between 10 µM [45,46,47] and 50 µM [41]. Thus, one can establish at a glance that the in vitro maximal inhibitions reported here are not of nutritional relevance and lie in the toxicological range since severe intoxications have been reported when 1 mM caffeine is reached in plasma and since the generally accepted acute LD_50_ values are in the range of 150–200 mg/kg [8]. As for the above-mentioned PrAO inhibition, it is not necessary to reach maximal inhibition of insulin signaling by any given ingested dietary compound to exert a modulatory influence on glucose disposal. Over a long time, the small limitation of glucose utilization by adipose tissue in post-prandial states may account for a portion of the anti-obesity effect of sustained caffeine ingestion or supplementation [48]. By comparison, the ‘’browning’’ action of caffeine, which increases non-shivering thermogenesis by transforming white preadipocytes into beige or brown adipocytes, and which has been proposed to mitigate obesity by enhancing energy dissipation, has been observed at 1.5–3 mM [49].

In keeping with the capacity of caffeine to impair the insulin activation of glucose metabolism by adipocytes, our attempt to extrapolate to humans was limited by the small sample size of subjects. We obtained less significant results in humans than in rodents. This limitation was likely due to a higher inter-individual variability of insulin sensitivity in humans than in animal models (compare Figure 4 and Figure 5). The present work, nevertheless, confirmed that caffeine prevents insulin-dependent glucose uptake in murine adipocytes as claimed in [11,12,13]. We demonstrated that this property also applies for human mature fat cells, but we could not clearly demonstrate that caffeine is antilipogenic in humans. Nevertheless, the effects of caffeine or IBMX observed in rodent fat cells are undoubtedly representative of those occurring in Man. The preclinical aspect of our work needs to be reinforced by more extensive studies with larger cohorts. It is required to determine whether a daily intake of caffeine below the safe limit of 400 mg per consumer [2,44] can really affect adipocytes in the manner proposed. Such future clinical studies should also characterize sensitive populations to take into account inter-individual variability in insulin and caffeine responsiveness.

Unfortunately, parallel functional explorations of lipolytic, glucose transport, and lipogenic responses in the same batch of adipocytes were not feasible. Thus, no quantitative analysis of the respective activation or inhibition of these functions by caffeine could be carried out. In other words, we could not clarify whether the inhibition of glucose transport, which is the first step of de novo lipogenesis, was sufficient and necessary for caffeine or IBMX to impair lipogenic activity in fat cells. Nevertheless, on the basis of the results obtained with AOC3KI mice, we can propose that PrAO inhibition is not required by caffeine to exhibit antilipogenic properties. These unexpected findings indicated that our working hypothesis was inadequate, as it is in the case of the suggestions of Papukashvili et al., who recently proposed caffeine as an effective, safe, and reliable choice to limit SSAO activity and thereby attenuating weight gain or diabetes [28,29]. Our observations only rule out a mediation of the antilipogenic effect of caffeine by direct inhibition of PrAO activity, and leave the exact mechanisms of this antilipogenic effect not well characterized. However, our findings do not call into question the anti-adipogenic effects of prolonged treatment with the methylxanthine, involving down-regulation of proadipogenic transcription factors such as C/EBPα and PPARγ [35,50], and the activation of fat browning [9,49], which may contribute to limit fat deposition in mice subjected to obesogenic diets [51] or in coffee consumers [8].

The rapid impairment of glucose utilization by caffeine reported here for the first time in mature human adipocytes is probably contributing to the increased blood glucose levels found after caffeine ingestion in numerous clinical studies (reviewed in [52]). However, a clinical study performed in patients with tetraplegia, who cannot release epinephrine in response to a central effect of caffeine, elegantly showed that when caffeine ingestion cannot release epinephrine, it does not impair glucose tolerance [53]. This indicated that hepatic glucose output, which is activated by adrenergic stimulation, can also contribute to the slight impairment of glucose tolerance by caffeine [52].

As caffeine was equally antilipogenic when tested at 0.1–1 mM in mature adipocytes isolated from AOC3KI and WT mice, the supposed contribution of PrAO inhibition in the caffeine-induced inhibition of glucose incorporation into lipids was ruled out. However, a potential explanation for the persistence of caffeine-dependent antilipogenesis action in the adipocytes from AOC3KI mice lies in the possibility of multiple proteins having a SSAO activity. However, we reported that benzylamine oxidation was totally abolished in the tissues of AOC3KI mice [32], as found in independent studies on the same model [30,31]. Although not being the preferential substrate for all the *AOC* gene products, benzylamine can be oxidized by various copper-containing amine oxidases and the abolishment of its oxidation does not leave much opportunity for compensatory upregulation of other amine oxidases in adipose tissue. Lastly, the caffeine antilipogenic action surprisingly found in AOC3KI mice led us to hypothesize that MAO inhibition was involved in lipogenesis impairment. As the reference MAO inhibitor pargyline did not dramatically impair insulin-stimulated lipogenesis in AOC3KI and WT, as well as in other mice [21,40], this eventuality was discarded.

It is also possible that a site, other than the active site, on the inactive AOC3KI PrAO protein could bind caffeine and mediate these effects. Imidazole binding sites on PrAO have been described [54] and may be involved in PrAO inhibition by caffeine [26]. On the other hand, the imidazole binding sites are close to the active site and it is difficult to conceive that they would be unaffected by perturbations in this region of the protein. Moreover, to date, the physiological role of PrAO in inflammation has been shown to require that it is catalytically active [31]. However, to distinguish between this possibility and the existence of other protein targets will require further detailed experiments. Nonetheless, it is clear from these data that the antilipogenic properties of caffeine do not depend on a catalytically active PrAO.

## 4. Materials and Methods

### 4.1. Reagents and Chemicals

Caffeine, 3-isobutyl-1-methylxanthine (IBMX), benzylamine, tyramine, amine oxidase inhibitors, and other reagents were from Sigma-Aldrich (Saint Quentin Fallavier, France), as well as bovine insulin and bovine albumin and other reagents. [^3^H]-2-deoxyglucose and d-[3-^3^H]-glucose were supplied by Perkin Elmer (Boston, MA, USA). Liberase was from Roche Diagnostics (Mannheim, Germany).

### 4.2. Animal Models

Normoglycemic Wistar rats (a total of 18 males of 200–300 g body weight) and C57BL/6 mice of both sexes were purchased at Charles River (L’Arbresle, France) for an interspecies comparative approach, with the approval of the Animal Ethics Committee of INSERM (code 12-1048-03-15). When indicated, AOC3KI mice genetically modified on the *Aoc3* gene, were used in parallel to their wild type control (WT) as previously described when studying responses in adipocytes lacking SSAO activity [32]. The mice of the knock-in lineage called AOC3KI were obtained after homologous recombination with a mutated *Aoc3* gene, encoding for a nonfunctional SSAO/PrAO/VAP-1, by genOway (Lyon, France). Offspring backcrossed onto a C57BL/6 genetic background were kindly donated by Dr D. Smith (BioTie Ther., Turku, Finland). No SSAO activity was found in tissues from homozygous knock-in mice, while activity was present in WT, as previously reported [30,32].

### 4.3. Patients and Adipose Tissue Biopsies

Subcutaneous abdominal adipose tissue biopsies were obtained from overweight or mildly obese women undergoing reconstructive surgery at Rangueil Hospital, Toulouse (France). After surgical excision, samples were transferred at INSERM Unit 1048 in less than 2 h. Samples of 1 to 2 g were immediately used for adipocyte isolation at 37 °C and the remaining tissue frozen at −80 °C. Among the subgroups of patients constituted for this study, the mean age was 43 years and the mean body mass index was 26.0 ± 0.9 kg/m^2^ (*n* = 18).

### 4.4. Preparations of Functional Adipocytes from Rodent and Human Adipose Depots

Functional assays for determining lipolytic activity, glucose transport, and lipogenic activities could not be carried out in parallel, since each exploration was performed on freshly isolated adipocyte suspensions. In rodents, the white adipose tissues (WAT) from subcutaneous and visceral regions were dissected and immediately digested with collagenase as previously described [36]. Once isolated, the buoyant adipocytes were resuspended in different media routinely used for exploring lipolysis, hexose uptake, and lipogenic activities [55]. The same procedure was applied to human adipocyte preparations, when the schedule and quantity of subcutaneous samples received from the surgery department were compatible with immediate digestion with 15 µg/mL liberase (Roche Diagnostics, Meylan, France), washing, filtration, and subsequent incubations. All adipocyte preparations were performed at 37 °C in Krebs–Ringer buffer pH 7.4, containing 15 mM bicarbonate, 10 mM HEPES, and 3.5% of bovine serum albumin (KRBH buffer). Glucose was omitted during these preparative steps, then used at 5.5 mM for lipolysis, 0.6 mM by isotopic dilution of [^3^H]-glucose for lipogenesis, while glucose was replaced by 0.1 mM [^3^H]-2-deoxyglucose plus 2 mM pyruvate for hexose transport assays, as previously stated [37,56].

### 4.5. Lipolysis and Lipogenesis Assessment in Rodent and Human Adipose Cells

Lipolysis was assessed by determining glycerol release under similar conditions as previously described [57] since we reported recently that the patterns of glycerol and FFA release were very similar in response to most lipolytic and antilipolytic agents [58]. In this set of experiments, the incubations lasted 120 min, for the sake of similarity to lipogenesis conditions, except for mouse adipocytes, for which glycerol release was determined after 90 min.

For lipogenesis, [^3^H]-glucose incorporation into lipids was measured via the original insulin bioassay using D-3-[^3^H]-glucose developed by Moody et al. [59] with slight adaptations [60]. Adipocyte lipids, including labeled ones generated by de novo lipogenesis during a 120 min incubation, were extracted using a non-water-miscible liquid scintillation cocktail designed for non-aqueous solutions (InstaFluor-Plus, PerkinElmer, Waltham, MA, USA). A lower aqueous and an upper organic phase were formed in the scintillation vials, but only the radiolabeled lipids present in the latter were counted since the tritium remaining in the lower phase, essentially in the form of non-metabolized glucose, could not excite the hydrophobic scintillation probe as detailed in [40,61]. For lipolysis and lipogenesis, results were expressed either per 100 mg cellular lipids or as a percentage of maximal stimulation, obtained with 10 µM isoprenaline or 100 nM insulin, respectively [21].

### 4.6. Glucose Transport Assay in Rodent and Human Adipose Cells

To investigate the effect of caffeine on glucose transport, the radiometric method based on the uptake of the non-metabolizable [^3^H]-2-deoxyglucose (2-DG) during 10 min incubation was used as previously described for human [62], rat [63], or mouse [36] fat cells. The tested agents, including caffeine, were incubated for 45 min with adipocytes in KRBH prior to addition of 2-DG.

### 4.7. Amine Oxidase Activities in Human Adipose Tissue

Thawed samples of human adipose tissue were homogenized for 30 s at room temperature to avoid the formation of a fat cake, which occurs at colder temperatures, and which is inappropriate for dispensing into assay tubes. Incubations were then performed for 30 min at 37 °C in a final volume of 200 µL, containing 200 mM phosphate buffer at pH 7.5 with or without inhibitors and substrates (tyramine and benzylamine). Hydrogen peroxide, which is one of the products of amine oxidation, was determined by a fluorometric method using the probe Amplex Red (from FluoProbes, InterChim, Montluçon, France), as described in [27].

### 4.8. Statistical Analysis

Results are presented as means ± SEM using GraphPad Prism 6 for Mac OSX by one-way ANOVA followed by Dunnett multiple comparisons. Student’s paired *t*-test was used when specified to compare to respective control. NS: denotes no significant difference.

## 5. Conclusions

The ability of caffeine to inhibit amine oxidases and to impair basal and insulin-stimulated glucose uptake and incorporation into lipids of adipocytes has to be added to its well-known lipolytic and thermogenic properties, indicating that caffeine and related methylxanthines are still of potential interest for the treatment or prevention of obesity. It deserves to be elucidated in future clinical trials whether sustained consumption or supplementation is beneficial to limit excessive fat deposition or adipose tissue inflammation associated with obesity complications.

## Data Availability

Data processed during the current study are available from the corresponding author upon reasonable request.

## Sample Availability

Samples of are not available from the authors.
